# Prognostic significance of necrosis in ampullary carcinomas

**DOI:** 10.1007/s11845-024-03740-3

**Published:** 2024-06-26

**Authors:** Kadriye Ebru Akar, Pelin Bagci

**Affiliations:** Department of Pathology, Marmara University School of Medicine, Istanbul, Turkey

**Keywords:** Adenocarcinoma, Ampulla of Vater, Carcinoma, Necrosis, Prognosis

## Abstract

**Background/aims:**

Necrosis is an important pathological feature that reflects high malignancy potential in tumors such as hepatocellular carcinoma and renal cell carcinoma. We aimed to elucidate the prognostic impact of necrosis in ampullary carcinomas.

**Materials and methods:**

We reviewed 101 consecutive cases of ampullary carcinoma for tumor necrosis, types of necrosis, macroscopic and microscopic histopathological subtypes, lymphatic-vascular-perineural invasions, and other histopathological parameters.

**Results:**

Tumor necrosis was present in 19 (18.8%) cases and was identified as an independent poor prognostic indicator in multivariate survival analysis (*p* = 0.029).

**Conclusion:**

The presence of necrosis in ampullary carcinomas is directly related to vascular and perineural invasion and is a poor prognostic indicator independent of tumor stage. Including the presence of necrosis in the pathology reports of ampullary carcinomas will facilitate risk stratification.

## Introduction

Ampullary carcinomas represent 0.5% of all malignancies in the gastrointestinal tract [1]. They typically have a better prognosis than other tumors in the periampullary region, primarily because they present with early symptoms [2]. Currently, there are several unfavorable prognostic factors for ampullary carcinomas, including pancreatobiliary histological type [3, 4], lymph node metastasis [5, 6], and positive resection margins [7, 8]. However, due to the variable survival times reported in the literature [9, 10], it is necessary to define better predictive factors that can accurately identify poor prognosis in ampullary carcinomas.

Tumor necrosis is a straightforward, nonspecific, yet easily assessable histopathological variable. It is recognized as a negative prognostic factor in various cancers including hepatocellular carcinoma, renal cell carcinoma, non-small cell lung cancer, breast cancer, pancreatic cancer, colon cancer, soft tissue sarcomas, and malignant mesotheliomas [11–17].

Our study aimed to document the presence and type of necrosis in ampullary carcinomas, investigate its relationship with other histomorphologic parameters, and determine its effects on survival.

## Materials and methods

From institutional archives, we retrieved 101 consecutive cases of ampullary carcinoma resections between 2013 and 2021. A uniform sampling method was employed for all resections, which included orange-peeling for lymph nodes [18, 19], and clockwise sampling around the ductular lumen for tumor assessment [18]. In each case, the entire tumor was sampled. Patients who received neoadjuvant therapy were excluded from the study. All hematoxylin and eosin (H&E)-stained slides underwent review, and two pathologists re-evaluated the following pathological factors: macroscopic subtypes, histological subtypes, tumor stage, lymph node metastasis, lymphatic/vascular/perineural invasion, and the presence and type of necrosis. Clinical and survival data were extracted from pathology reports and clinical records.

**Fig. 1:**
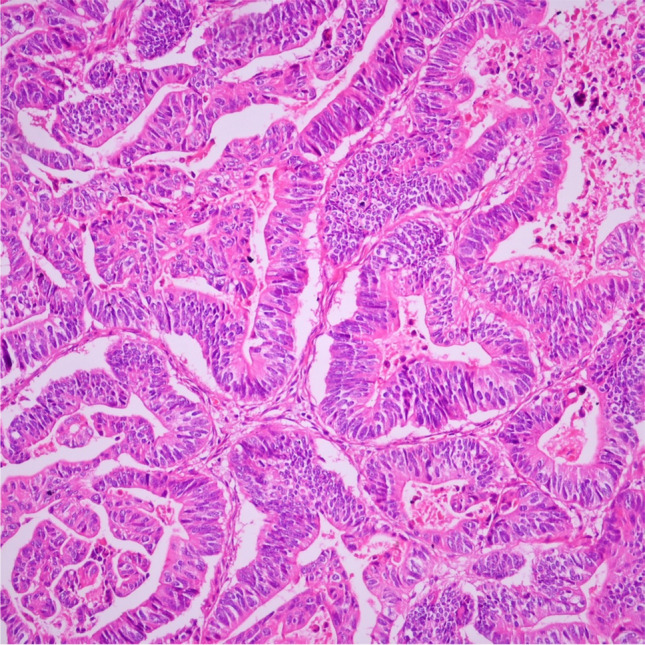
Intestinal histomorphology (H&E, × 200)

**Fig. 2:**
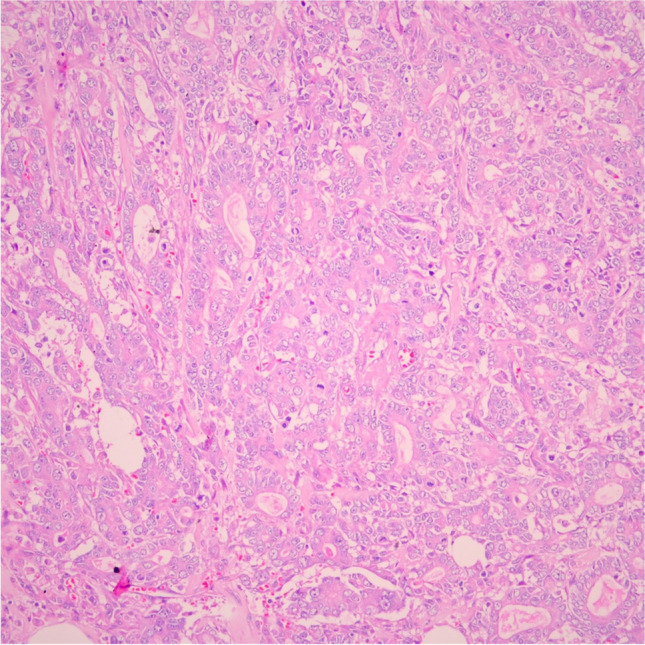
Pancreatobiliary histomorphology (H&E, × 200)

Macroscopically, tumors were classified into four subgroups based on their localization, as defined in the literature: ampullary ductal, intra-ampullary papillary tubular neoplasm (IAPN)-associated, periampullary duodenal, and ampullary-NOS [7]. The microscopic type was determined based on predominant morphologic and cytologic features. Accordingly, tumors were classified as “intestinal-type” if predominantly characterized by branching and interconnected tubules with columnar-shaped and pseudostratified tumor cells (Fig. 1). Tumors were classified as “pancreatobiliary-type” if predominantly characterized by scattered, small, well-formed tubules with 1- to 2-cell layers of more cuboidal nuclei (Fig. 2). Non-tubular tumors that could not be classified into these two groups were grouped as “other types.”

**Fig. 3:**
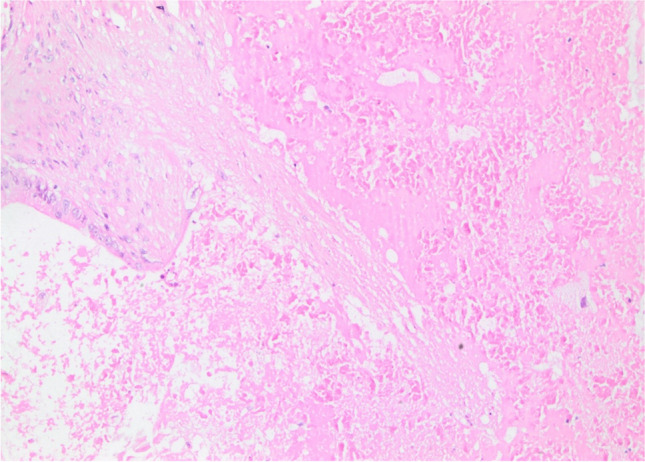
Ischemic/geographical necrosis (H&E, × 200)

**Fig. 4:**
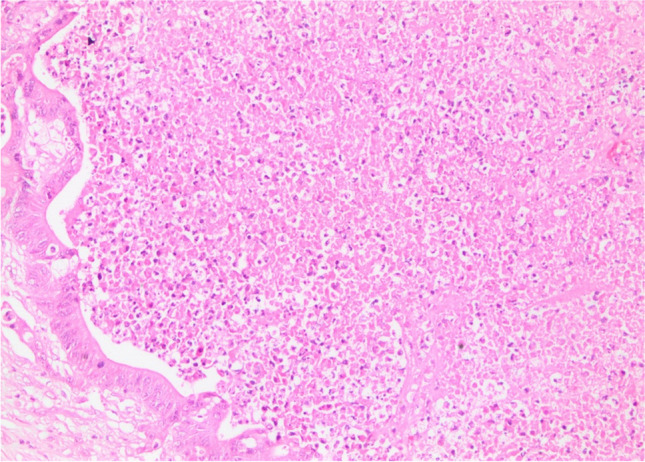
Dirty necrosis (H&E, × 200)

Necrosis characterized by homogeneous clusters with only persisting cell borders was classified as ischemic or geographical necrosis (Fig. 3). Necrosis containing inflammatory cells and/or inflammatory debris alongside persisting cell borders was categorized as dirty necrosis [20, 21] (Fig. 4). If a case included both types of necrosis, we considered the necrosis type as combined necrosis.

Statistical analyses were conducted using the Statistical Package for the Social Sciences version 26.0 (SPSS) and the Jamovi Module [22]. Descriptive analyses were presented using mean and median (min–max) for numerical variables. Categorical variables were examined using the Pearson chi-square test, Yates chi-square test, and Fisher’s exact test. Overall survival (OS) rates were estimated using the Cox proportional-hazards regression model. A *p*-value of < 0.05 was considered statistically significant.

## Results

The study included 59 males (58.4%) and 42 females (41.6%). The mean age was 64 years (range 39–84 years). Patients underwent pancreaticoduodenectomy (*n* = 93, 92.1%) or ampullectomy (*n* = 8, 7.9%). The median follow-up period was 37.5 months (range 1–116 months).

The tumors were divided into three groups based on their largest diameters: 45 (44.6%) were < 2 cm, 45 (44.6%) were 2–4 cm, and 11 (10.9%) were > 4 cm. The median tumor size was 2.37 cm. Lymphatic invasion, vascular invasion, and perineural invasion were observed in 89 (88.1%), 65 (64.4%), and 61 (60.4%) cases, respectively. T stages were re-classified according to the current cancer protocols (College of American Pathologists 2021 guidelines): 26 (25.7%) patients were classified as stage T1a + 1b; 20 (19.8%) as stage T2; and 55 (54.5%) as T3a + 3b. Sixty-five patients had lymph node metastasis (stage N1, 35 (37.6%); stage N2, 30 (32.3%)]. In 8 ampullectomy materials, lymph nodes could not be examined. Resection margins were involved in 19 (18.8%) cases, all of which were positive for retroperitoneal margins, and one was also positive for pancreatic distal margin and common bile duct margin (Table 1).

**Table 1:** Clinicopathological features of the cases and their relationship with necrosis

Variables	Groups	*n*	%	Necrosis present (*n*/%)	Necrosis absent (*n*/%)	*p* value
Gender	F–M	42/59	41.6/58.4	13/31–6/10.2	29/69–53/89.8	0.018*
Age	(< 65– ≥ 65)	45/56	44.6/55.4	7/15.6–12/21.4	38/84.4–44/78.6	0.621
Tumor size	< 2 cm	45	44.6	3/6.7	42/93.3	0.009*
	2–4 cm	45	44.6	11/24.4	34/75.6	
	> 4 cm	11	10.9	5/45.5	6/54.5	
Macroscopic type	Ampullary-ductal	50	49.5	7/14	43/86	0.018*
	IAPN-associated	39	38.6	6/15.4	33/84.6	
	Periampullary-duodenal	8	7.9	5/62.5	3/37.5	
	Ampullary-NOS	4	4	1/25	3/75	
Microscopic type	Pancreatobiliary	69	68.3	12/17.4	57/82.6	0.798
	Intestinal	16	15.8	4/25	12/75	
	Other	16	15.8	3/18.8	13/81.2	
Lymphatic invasion	Present	89	88.1	19/21.3	70/78.7	0.116
	Absent	12	11.9	0/0	12/100	
Vascular invasion	Present	65	64.4	17/26.2	48/73.8	0.023*
	Absent	36	35.6	2/5.6	34/94.4	
Perineural invasion	Present	61	60.4	16/26.2	45/73.8	0.036*
	Absent	40	39.6	3/7.5	37/92.5	
Lymph node status	Positive	65	69.9	14/21.5	51/78.5	0.993
	Negative	28	30.1	5/17.9	23/82.1	
Resection margin	Positive	19	18.8	5/26.3	14/73.7	0.345
	Negative	82	81.2	14/17.1	68/82.9	
N stage	N0	28	30.1	5/17.9	23/82.1	0.653
	N1	35	37.6	9/25.7	26/74.3	
	N2	30	32.3	5/16.7	25/83.3	
T stage	T1a + T1b	26	25.7	0/0	26/100	0.005*
	T2	20	19.8	5/25	15/75	
	T3a + T3b	55	54.5	14/25.5	41/74.5	

Values written in bold and with an asterisk indicate statistically significant values

Macroscopically, among 101 ampullary carcinoma cases, 50 (49.5%) were ampullary-ductal, 39 (38.6%) were IAPN-associated, 8 (7.9%) were periampullary-duodenal, and 4 (4%) were ampullary-NOS. Histologically, 69 cases (68.3%) were pancreatobiliary-predominant, 16 cases (15.8%) were intestinal-predominant, and 16 cases (15.8%) were other types (9 poorly cohesive carcinomas, 5 mucinous carcinomas, and 2 squamous cell carcinomas) (Table 1).

Necrosis was observed in 19 (18.8%) cases; among them, 11 (57.9%) exhibited “dirty necrosis,” 1 (5.3%) had “geographical/ischemic necrosis,” and 7 (36.8%) displayed “combined necrosis” (both necrosis types). Among the pancreatobiliary-dominant tumors, 7 (10.1%) had dirty necrosis, 1 (1.4%) had geographical necrosis, and 4 (5.8%) had combined necrosis; whereas among the intestinal-dominant tumors, 2 (12.5%) had dirty necrosis, and 2 (12.5%) had combined necrosis. Among other histologic groups, 2 (12.5%) cases had dirty necrosis, and 1 (6.3%) had combined necrosis (Table 2).

**Table 2:** Distribution of necrosis types among histomorphological tumor types

Necrosis type	Dirty (*n*/%)	Ischemic/geographical (*n*/%)	Combined (*n*/%)
Pancreatobiliary type	7/10.1	1/1.4	4/5.8
Intestinal type	2/12.5	0/0	2/12.5
Other histologic types	2/12.5	0/0	1/6.3

### The association between necrosis, clinicopathological parameters, and survival

**Fig. 5:**
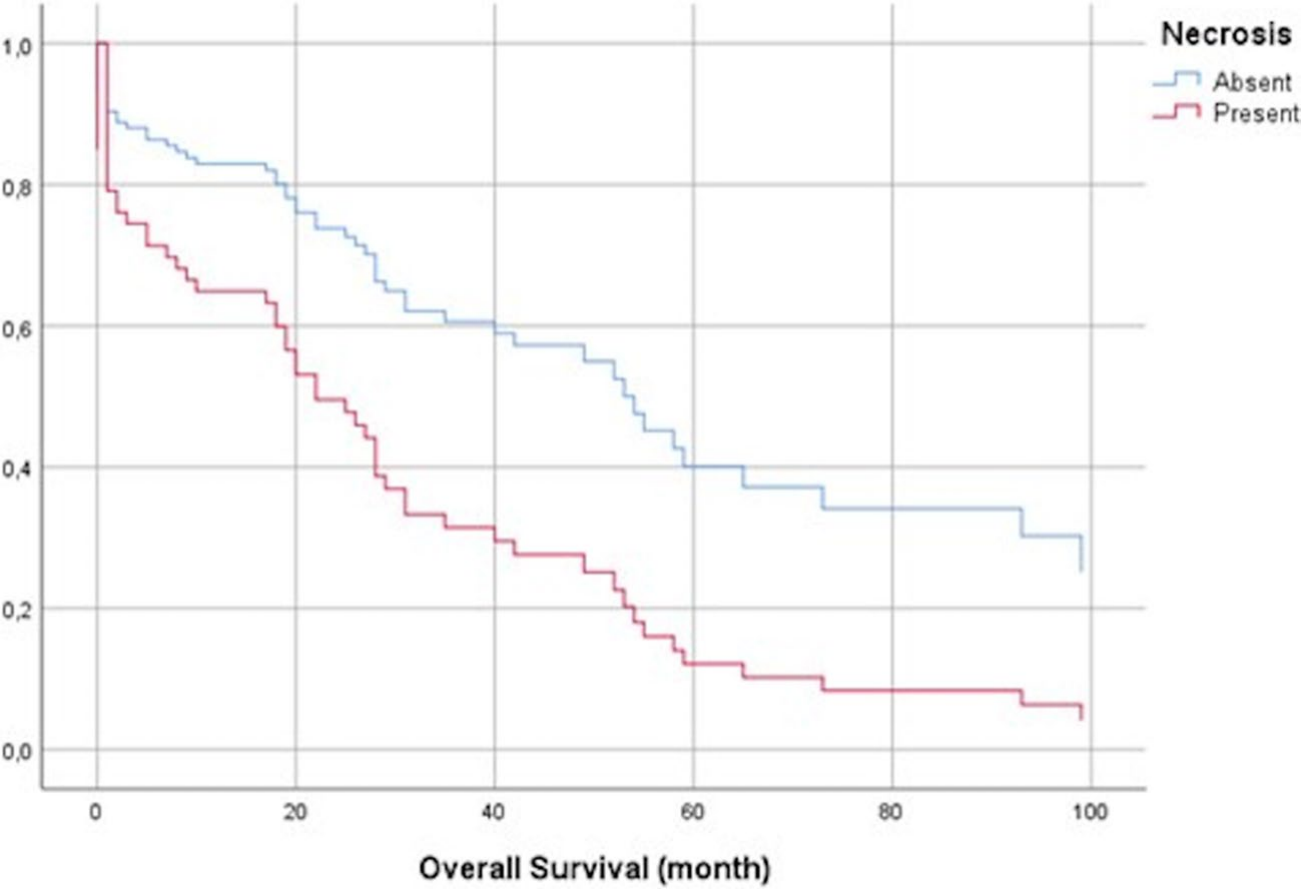
Cox regression survival curve of necrosis for overall survival

In univariate survival analysis, the presence of necrosis, age, microscopic type, N stages, lymph node metastasis, and resection margin status was identified as statistically significant poor risk factors for overall survival (*p* values 0.030, 0.017, 0.001, 0.005, 0.039, 0.020, respectively) (Table 3). However, in multivariate analysis, only necrosis (Fig. 5) and age were found to be independent prognostic variables (*p* values 0.029 and 0.043, respectively) (Table 4).

**Table 3:** Univariate survival analysis

Variables	Groups	HR	95% Cl	*p* value
Gender	Female vs. male	1.258	0.761–2.080	0.372
Age	≥ 65 vs. < 65	1.886	1.119–3.179	0.017*
Tumor size	< 2 cm	Ref		
	2–4 cm	1.131	0.668–1.916	0.646
	> 4 cm	1.247	0.513–3.030	0.626
Macroscopic type	Ampullary-ductal	Ref		
	IAPN-associated	0.765	0.446–1.312	0.331
	Periampullary-duodenal	0.749	0.265–2.115	0.585
	Ampullary-NOS	0.681	0.163–2.842	0.598
Microscopic type	Pancreatobiliary	Ref		
	Intestinal	0.456	0.194–1.075	0.073
	Others	2.675	1.336–5.357	0.005*
Necrosis	Present vs. absent	1.930	1.067–3.489	0.030*
Necrosis type	Dirty	Ref		
	Ischemic/geographical	1.472	0.177–12.217	0.720
	Combined	1.006	0.346–2.925	0.991
Lymphatic invasion	Present vs. absent	2.220	0.885–5.565	0.089
Vascular invasion	Present vs. absent	1.730	0.996–3.006	0.052
Perineural invasion	Present vs. absent	1.402	0.825–2.382	0.211
Lymph node status	Positive vs. negative	1.969	1.034–3.750	0.039*
Resection margin	Positive vs. negative	2.020	1.117–3.653	0.020*
N stage	N0	Ref		
	N1	1.348	0.664–2.739	0.409
	N2	2.776	1.396–5.519	0.004*
T stage	T1a + T1b	Ref		
	T2	0.794	0.360–1.751	0.567
	T3a + T3b	1.240	0.685–2.244	0.477

**Table 4:** Multivariate survival analysis

Variables	Groups	HR	95% Cl	*p* value
Age	≥ 65 vs. < 65	1.842	1.021–3.324	0.043*
Microscopic type	Pancreatobiliary	Ref		
	Intestinal	0.671	0.275–1.637	0.380
	Others	1.848	0.848–4.027	0.122
Necrosis	Present vs. absent	2.061	1.075–3.949	0.029*
Lymph node status	Positive vs. negative	3.804	0.462–31.343	0.214
Resection margin	Positive vs. negative	1.141	0.543–2.397	0.728
N stage	N0	Ref		
	N1	0.351	0.044–2.773	0.321
	N2	0.692	0.088–5.432	0.726

**Fig. 6:**
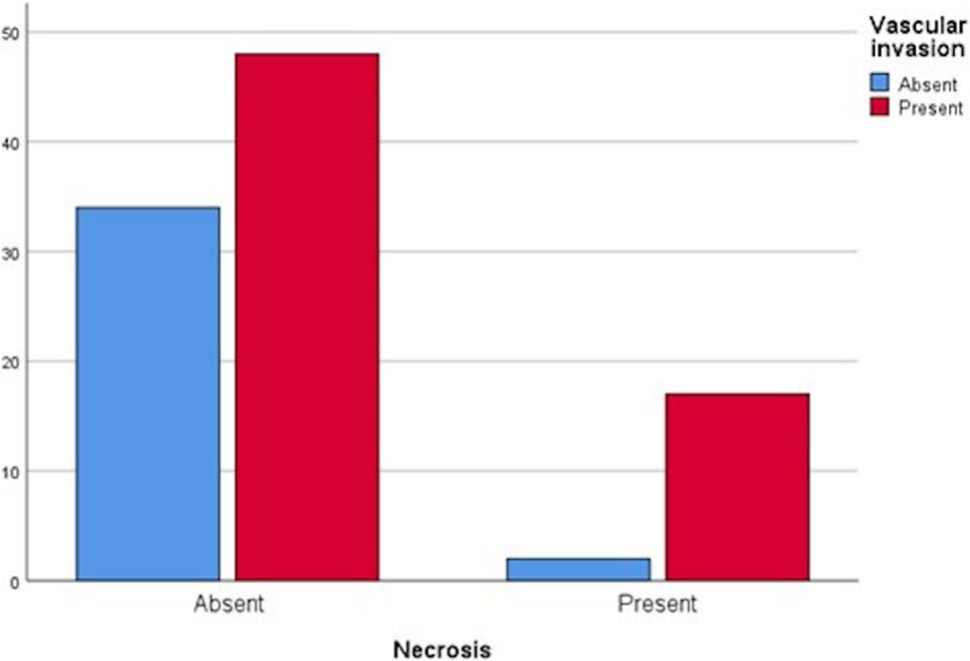
Relationship between necrosis and vascular invasion

Out of 101 cases, 19 (18.8%) had necrosis. When cases with and without necrosis were compared, the female/male ratio (2.16 with necrosis vs. 0.54 without necrosis) was significantly higher, and there were higher rates of vascular invasion (Fig. 6) and perineural invasion in the group with necrosis (*p* values 0.018, 0.023, 0.036, respectively). Additionally, necrosis rates were statistically higher in cases with larger tumor sizes, periampullary-duodenal macroscopic types, and advanced tumor stages (*p* values 0.009, 0.018, and 0.005, respectively (Table 1).

Age ≥ 65 years, microscopic type, lymphatic invasion, presence of positive lymph node, positive surgical margin, and N stage parameters were not significantly different between the groups with and without necrosis (*p* values 0.621, 0.798, 0.116, 0.993, 0.345, 0.653, respectively) (Table 1).

 In the assessment of necrosis type, dirty necrosis was predominant, occurring in the majority of cases (11 out of 19), while ischemic/geographical necrosis was observed in only one case. Additionally, combined necrosis was present in 7 cases. When necrosis types were compared with other histologic parameters, no significant results were observed for gender, age, tumor size, macroscopic type, microscopic type, lymphatic invasion, vascular invasion, perineural invasion, lymph node metastasis, resection margin status, N stage, and T stage (*p* values 0.404, 0.578, 0.247, 0.499, 1.00, N/A, 0.070, 1.00, 0.702, 1.00, 0.917, 0.702, respectively).

## Discussion

The present study demonstrates that tumor necrosis is a stage-independent negative prognostic marker for ampullary carcinomas. Previously, several studies have reported that tumor necrosis is related to poor outcomes in patients with breast, renal, pancreatic, liver, lung, and colorectal malignancies [14, 23–28]. Although several histopathological parameters, including lymph node metastasis [5, 6], pancreatobiliary morphology [3, 4], lymphovascular invasion [5, 6], perineural invasion [6, 29], and positive margin status [7, 8], provide independent prognostic information according to other studies, prognostic stratification in ampullary carcinomas remains uncertain. Therefore, it is essential to describe new parameters that will provide strong prognostic stratification.

In contrast to other solid organ carcinomas, only one study has focused on the relationship between necrosis and survival in ampullary carcinomas [4]. In accordance with the study conducted by Carter et al., tumor necrosis showed no association with survival in either univariate or multivariate analysis. Furthermore, this study did not assess the relationship between the type of necrosis and other parameters.

 In a study by Kuroe et al. evaluating the prognostic effects of necrosis in renal cell carcinoma, similar to our study, it was reported that the presence of necrosis was an independent indicator of survival and was also associated with increased tumor size, advanced T stage, and vascular invasion. Furthermore, in the same study, necrosis types were analyzed in two categories: “dirty” and “ghost” (defined as ischemic/geographical necrosis in our study) necrosis. It was demonstrated that serum CRP levels, distant metastasis frequency, nuclear grade, and sarcomatoid differentiation were higher in “dirty” necrosis compared to “ghost” necrosis [21].

In our study, no significant correlation was observed between necrosis types and other parameters, primarily due to the limited number of patients presenting with dominant-ischemic necrosis. However, it is worth noting that previous research has identified an association between necrosis and aggressive tumor parameters in pancreatic adenocarcinomas [14, 30]. According to these studies, aggressive progression parameters such as vascular invasion, metastasis, higher TNM stage, larger tumor size, and higher histological grade were closely related to intratumoral hypoxia and necrosis. Intratumoral hypoxia is a mechanism created through HIF-1α (hypoxia-induced factor-1α), which is known to induce tumor dedifferentiation, rapid growth, invasion, metastasis, angiogenesis, and resistance to chemotherapy, resulting in the formation of aggressive tumor morphology [31, 32]. Since the morphological reflection of intratumoral hypoxia is necrosis, the detection of necrosis is accepted as an indicator of aggressive tumor behavior. In the study by Hiraoka et al., the Carbonic Anhydrase IX (a transmembrane protein regulated by hypoxia) immunohistochemical stain showed concentrated expression around areas of necrosis, demonstrating the relationship of necrosis with intratumoral hypoxia [30]. In our study, necrosis was associated with increased tumor size, vascular invasion, perineural invasion, and advanced T stage, thus supporting the hypothesis of intratumoral hypoxia.

Besides the detailed observation of necrosis and other histological parameters and their correlation with prognosis, our study has some limitations. Firstly, it was retrospective and conducted at a single center. Secondly, the sample size was relatively small, limiting the scope for definitive analysis, and should be tested in larger cohorts.

In conclusion, tumor necrosis facilitates risk stratification of ampullary cancers and serves as an independent prognostic factor. The presence and type of necrosis are associated with an increased risk of poor outcomes and should be separately reported in synoptic reports. Additionally, the presence and type of necrosis can be valuable, particularly in small endoscopic or endoscopic ultrasound (EUS)-guided fine-needle aspiration (FNA) biopsies, aiding in distinguishing between dysplasia and/or invasive carcinoma, as well as determining the type of invasive carcinoma (pancreatobiliary vs. intestinal). For non-operable cases, this endoscopic subtyping is crucial in decision-making regarding chemotherapy options. Moreover, including the presence of necrosis as a parameter in ampullary carcinoma reports may facilitate the evaluation of potential HIF1α-targeting or antiangiogenesis therapies that can be used in these patients.

## Data Availability

Data supporting this study are included within the article and/or supporting materials.
